# Can delta neutrophil index values predict the success of periodontal treatment in patients with periodontitis?

**DOI:** 10.1007/s00784-023-05478-1

**Published:** 2024-01-09

**Authors:** Eda Çetin Özdemir, Esra Bozkurt, Fatih Mehmet Yazar, Mehmet Buğra Bozan

**Affiliations:** 1 Department of Periodontology, Faculty of Dentistry, Kahramanmaraş Sütçü İmam University, Kahramanmaraş, 46000 Turkey; 2 Department of General Surgery, Private Sular Akademi Hospital, Kahramanmaraş, Turkey; 3 Department of General Surgery, Faculty of Medicine, Kahramanmaraş Sütçü İmam University, Kahramanmaraş, Turkey

**Keywords:** Delta neutrophil index, Immature granulocytes, Non-surgical periodontal therapy, Periodontitis

## Abstract

**Objectives:**

The aim of this study was to evaluate the effect of delta neutrophil index (DNI) on non-surgical periodontal therapy (NSPT), whose role has been documented in the pathogenesis and follow-up of periodontal disease.

**Methods and materials:**

The study included 35 patients with stage 3, grade A periodontitis (test group) and 35 patients without periodontal disease (control group). Initially, periodontal parameters were recorded and blood samples were taken from all patients. For patients with periodontitis, periodontal parameter measurements and blood sample analyses were repeated 3 months after NSPT.

**Results:**

After NSPT, DNI, CRP (C-reactive protein), neutrophil count, WBC (white blood cell), and neutrophil–lymphocyte ratio (NLR) values decreased in the test group, but did not reach a statistically significant level (*p* > 0.05). When the inflammatory variables were examined, significantly higher CRP, IG (immature granulocytes), DNI, neutrophil count, and WBC were observed in the test group compared to the control group (*p* < 0.05). In the test group, periodontal parameters were lower 3 months after NSPT than at baseline (*p* < 0.05).

**Conclusion:**

Consistent with previous findings in the literature, the patients with periodontitis were determined to have higher levels of DNI, CRP, neutrophils, and WBC, compared to the individuals without periodontitis. Although a decrease was seen in DNI after NSPT, this was not at a significant level.

**Clinical relevance:**

DNI is a guide in the evaluation of inflammation at the onset of periodontal disease, but studies with a larger number of cases are needed to use these parameters in the evaluation of treatment success.

**Trial registration:**

This study was retrospectively registered on December 27, 2022, with the number NCT05666622 at http://www.clinicaltrials.gov.

**Supplementary Information:**

The online version contains supplementary material available at 10.1007/s00784-023-05478-1.

## Introduction

Periodontitis is a multifactorial chronic inflammatory disease that develops because of dysbiosis between the plaque biofilm and the host and is characterized by the destruction of the supporting tissues of the tooth. Clinical attachment loss due to support tissue destruction, radiographically visible alveolar bone loss, periodontal pocket formation, and gingival bleeding are the main clinical features of periodontitis [1]. Periodontitis is classified according to a versatile staging and grading system in the new classification introduced at the workshop held in 2017 [2]. Periodontitis stages are a simple description of the severity and complexity of the disease, while periodontitis grades describe the risk of progression and risk factor profile [2]. The pathological process of periodontitis begins with the accumulation of dental plaque biofilm and below the gingival margin; this condition becomes increasingly dysbiotic, resulting in dysregulation of the host immune-inflammatory response. This further increases dysbiosis and causes the destruction of periodontal tissues [3].

Microorganisms and microbial products found in biofilms trigger a local inflammatory response, and together with a systemic inflammatory response, the integrity of the periodontium is affected in stages [4]. Inflammatory mediators released from the periodontal tissues can activate the immune system, and a systemic acute phase response can be triggered [5]. Recent studies have reported that some hematological parameters that are pro-inflammatory mediators such as the neutrophil–lymphocyte ratio (NLR) [6], thrombocyte-lymphocyte ratio (PLR) [7], C-reactive protein (CRP) level [8, 9], neutrophil [10], and thrombocyte counts [11] are increased in patients with periodontitis and these parameters are associated with the severity of periodontitis [12].

The delta neutrophil index (DNI) is a parameter which is calculated as the ratio of immature granulocytes (IG) in peripheral circulation to the total neutrophil count [13]. The predictive value of DNI for infection and prognosis has been reported to be greater than that of traditional markers, including white blood cell (WBC) count, absolute neutrophil count, and CRP [14, 15]. In addition, a significant correlation has been shown between DNI and the severity of other infectious or inflammatory diseases such as rhinosinusitis [16], sepsis [17], acute appendicitis [18], piyelonefrit [19], and pancreatitis [20]. Therefore, it has been recommended in many studies that the use of DNI is of clinical benefit as a determinant or marker for early diagnosis, the decision for surgery, and prognosis in patients with various inflammatory and infectious diseases [17, 20, 21]. There is only one recent study, conducted by the current study authors, which has evaluated the potential role of DNI in the pathogenesis and follow-up of periodontal disease, and the results showed that there was a relationship between an increase in DNI and periodontal disease [21]. However, there is no study in the literature that has evaluated the effect of periodontal treatment on the level of DNI.

The aim of this study was to evaluate the potential role of DNI as a marker of periodontal disease in addition to the classic markers of NLR, CRP, procalcitonin, neutrophil count, and lymphocyte count in patients with stage 3, grade A periodontitis before and after non-surgical periodontal therapy (NSPT). The null hypothesis of this study is that no significant difference was found in inflammatory biomarkers and DNI levels in serum samples at the 3rd month after NSPT in patients with stage 3 grade A periodontitis.

## Method and materials

### Study design

All the procedures in this study were applied in compliance with the principles of the 2013 Helsinki Declaration. Approval for the study was granted by the Clinical Research Ethics Committee of Kahramanmaraş Sütçü Imam University (protocol no. 2020/348). The Strengthening the Reporting of Observational Studies in Epidemiology (STROBE) guidelines were followed [22]. Verbal and written informed consent were obtained from all the study participants.

### Patient selection

This prospective, clinical pilot study included patients under follow-up in the Periodontology Department of the Dentistry Faculty of Kahramanmaraş Sütçü Imam University between 1 October 2020 and 31 June 2021. Periodontal healthy (control group) and stage 3 grade A periodontitis (case group) patients were included in the study. Patients were excluded from the study if they had any systemic disease, were active smokers, were pregnant or breastfeeding, had used any antibiotic or anti-inflammatory drug in the last 6 months, had received any periodontal treatment in the last 6 months, or had fewer than 20 teeth.

As a result of clinical examinations and radiographic examinations, the systemically healthy participants were separated into 2 groups according to the 2017 World Periodontal and Peri-implant Diseases and Conditions Classification Workshop [23]. The control group was composed of 35 patients with good periodontal health, clinically healthy gingiva, no history of periodontitis, no attachment loss, no radiographic findings of alveolar bone destruction, and PD measured as ≤ 3 mm and BOP < 10%. The test group was composed of 35 patients with stage 3 periodontitis, with radiographic bone loss extending to the center or beyond the root, PD ≥ 6 mm, and interdental CAL ≥ 5 mm.

The patients determined with stage 3 periodontitis reported the loss of ≤ 4 teeth because of periodontitis. Evaluation of the extent and distribution of the disease showed CAL ≥ 5 mm in ≥ 30% of the teeth. The degree of bone loss/age was determined radiographically to estimate the progression of periodontitis [2]. All the patients were evaluated as grade A as the bone loss%/age was < 0.25.

A total of 82 patients were initially enrolled in the study. Following the exclusion of 7 patients who did not wish to participate because they did not want to give a blood sample, and 5 who did not attend the 3-month follow-up examination, the study was completed with 35 healthy control patients without periodontitis and 35 patients with stage 3, grade A periodontitis.

### Power analysis

G Power 3.0.10 (University Kiel, Germany) software was used to calculate the effect size. Due to a lack of studies regarding the delta neutrophil index in the periodontal treatment, Cohen’s *d* was accepted as 0.5 (medium) for the Wilcoxon rank test. A total of 35 was required for each group with 80% power and 0.5 type 1 error.

### Clinical measurements

Clinical measurements were made at the beginning of the study in all patients included in the test and control groups. Clinical measurements of the test group with stage 3 grade A periodontitis were repeated at 3 months after NSPT. The periodontal parameters of clinical attachment level (CAL), pocket depth (PD), gingival index (GI) [24], plaque index (PI) [25], and bleeding on probing (BOP) [26] were evaluated. Measurements were taken of each tooth from 6 regions (mesiobuccal, mid-buccal, distobuccal, distolingual/palatinal, mid-lingual/palatinal, and mesiolingual/palatinal) using a pre-calibrated manual Williams periodontal probe (Hu-Friedy, Chicago, USA). Clinical measurements were made by a single clinician (EÇÖ). The patients were also evaluated in respect of whether they had lost any teeth because of periodontitis. To provide calibration of the researcher, the periodontal clinical parameters of CAL and PD were measured twice at a 1-h interval in 5 volunteers. The first and second measurements were made blind and at least 90% repeatability was obtained with a mean difference of 1 mm.

### Non-surgical periodontal treatment

The patients included in the study were first informed that dental plaque is the main cause of periodontal diseases and about methods for removing plaque. Tooth brushing technique with the modified Bass technique was demonstrated to all patients on the model, and then, the use of the interdental brush and dental floss was explained. While no periodontal treatment was applied to the control group, patients in the test group were treated using ultrasonic devices (WOODPECKER® UDS-A Cavitron, Guilin Woodpecker Medical Ins. Co., China) and Gracey curettes (Gracey, SAS 5/6, SAS 7/8, SAS 11/12, SAS 13/14, Hu-Friedy, Chicago, IL, ABD) in 4 sessions. In the periodontitis group, in the first session, after full-mouth supragingival scaling, subgingival scaling and root planning were performed under local anesthesia (3 Ultra Cain® D-S forte, Sanofi-Aventis Deutschland GmbH, Germany), starting from a selected quadrant. A rubber brush was attached to the end of the rotary tool and polishing was carried out with the help of polishing paste at the end of each session. During NSPT, patients were not given any antibiotics or antimicrobial drugs. Oral hygiene training was repeated every session. Serum samples of periodontitis patients whose treatments were completed were taken at the 3rd month after NSPT. After clinical parameter measurements of the patients whose oral hygiene levels were checked, supragingival debridement and polishing procedures were performed when necessary. After the study follow-up period was completed, surgical periodontal treatments were applied to the areas deemed necessary in the evaluation.

### Serum sampling and study variables

Initially, venous blood samples were obtained from all participants from the antecubital vein into tubes with EDTA. The measurements of NLR, CRP, neutrophil count, procalcitonin, lymphocyte count, and DNI were performed using a calibrated automatic hematology analyzer (XN 3000, Sysmex Corpn, Japan). The NLR was calculated manually as neutrophil count/lymphocyte count, and the DNI was calculated as the ratio of IG to WBC [27]. In patients with stage 3 grade A periodontitis, 3 months after NSPT, blood samples were taken again with the same method, and the same parameters were evaluated.

### Statistical analysis

Data obtained in the study were analyzed statistically using the Jamovi software (Version 2.2.5). Normality of data distribution was checked with the Shapiro–Wilk test. Due to non-normal distribution, the Wilcoxon signed-rank test and the Mann–Whitney *U* test were conducted for intra-group and inter-group comparisons, respectively. The relationships between periodontal and inflammatory variables were analyzed with the Spearman correlation test. A value of *p* < 0.05 was accepted as statistically significant.

## Results

The age and gender distribution of the participants included in the study is shown in Table 1. The comparisons of inflammatory and clinical periodontal parameters within and between groups are shown in Table 2. No significant difference was observed between the inflammatory variables measured at baseline and at 3 months after NSPT in the test group (*p* > 0.05). In the test group, PI, GI, PD, BOP, and CAL values were lower at 3 months after NSPT than at baseline (*p* < 0.05). In the comparison of the inflammatory variables between the groups, the CRP, IG, DNI, neutrophil count, and WBC were significantly higher in the test group than in the control group (*p* < 0.05). In the comparison of periodontal parameters between the groups, the PI, GI, SCD, BOP, and CAL values were determined to be significantly higher in the test group than in the control group (*p* < 0.05). At 3 months after NSPT, the inflammatory variables of CRP, IG, DNI, neutrophil counts, and WBC values were significantly higher in the test group compared to the control group (*p* < 0.05).

**Table 1:** Age and gender distribution of the study participants

Demographic variables	Group	
	Control (*n* = 35)	Case (*n* = 35)
Age (years)	38.43 ± 7.01	37.14 ± 12.03
Sex (male/female) *n*	17/18	16/19

**Table 2:** The presentation of the intragroup (Wilcoxon signed-rank test) and intergroup (Mann–Whitney *U* test) comparisons

		Initial				3-month follow-up		
		*n*	Mean	Median	*n*	Mean	Median	*p*-value
Inflammatory variables								
CRP	Case	35	5.13 ± 3.25	3.96 (1.42–16.7)	35	5.06 ± 3.32	3.3 (2.21–15)	0.787^1^
	Control	35	3.43 ± 1.37	2.98 (1.42–9)	35	3.43 ± 1.37	2.98 (1.42–9)	
	*p*-value	0.004^2^				0.131^2^		
Prc	Case	35	0.05 ± 0.06	0.04 (0.02–0.33)	35	0.04 ± 0.03	0.03 (0.02–0.1)	0.452^1^
	Control	35	0.05 ± 0.02	0.04 (0.02–0.1)	35	0.05 ± 0.02	0.04 (0.02–0.1)	
	*p*-value	0.529^2^				0.158^2^		
IG	Case	35	0.04 ± 0.02	0.03 (0.01–0.11)	35	0.04 ± 0.03	0.03 (0.01–0.12)	0.352^1^
	Control	35	0.02 ± 0.01	0.02 (0.01–0.03)	35	0.02 ± 0.01	0.02 (0.01–0.03)	
	*p*-value	< .001^2^				< .001^2^		
DNI	Case	35	0.43 ± 0.24	0.4 (0.1–1.1)	35	0.37 ± 0.28	0.3 (0.02–1.2)	0.287^1^
	Control	35	0.23 ± 0.08	0.2 (0.1–0.4)	35	0.23 ± 0.08	0.2 (0.1–0.4)	
	*p*-value	< .001^2^				0.028^2^		
Neu	Case	35	8.84 ± 15.04	5.17 (2.47–69.1)	35	5.11 ± 1.33	4.77 (2.66–7.99)	0.914^1^
	Control	35	3.97 ± 1.01	3.82 (2.45–6.8)	35	3.97 ± 1.01	3.82 (2.45–6.8)	
	*p*-value	0.005^2^				< .001^2^		< .001^2^
Neu%	Case	35	60.29 ± 6.81	60.45 (48.5–72.2)	35	55.48 ± 14.78	56.65 (5.05–70.4)	0.364^1^
	Control	35	56.9 ± 7.38	57.9 (40.1–71.7)	35	56.9 ± 7.38	57.9 (40.1–71.7)	
	*p*-value	0.125^2^				0.737^2^		
Lym	Case	35	2.45 ± 0.72	2.34 (1.37–4.48)	35	2.43 ± 0.58	2.33 (1.4–3.64)	0.604^1^
	Control	35	2.27 ± 0.52	2.24 (1.39–3.27)	35	2.27 ± 0.52	2.24 (1.39–3.27)	
	*p*-value	0.457^2^				0.263^2^		
Lym%	Case	35	29.29 ± 6.86	28.25 (15.1–40.9)	35	29.64 ± 5.63	30.05 (18.6–37)	0.658^1^
	Control	35	32.99 ± 6.63	32.7 (19.6–48.2)	35	32.99 ± 6.63	32.7 (19.6–48.2)	
	*p*-value	0.077^2^				0.083^2^		
Plt	Case	35	265.8 ± 37.33	265 (206–342)	35	270.23 ± 40.32	267.5 (206–354)	0.564^1^
	Control	35	260.89 ± 65.6	258 (155–427)	35	260.89 ± 65.6	258 (155–427)	
	*p*-value	0.533^2^				0.392^2^		
WBC	Case	35	8.36 ± 2.04	8.07 (4.63–11.37)	35	7.95 ± 1.64	8.09 (4.7–12.5)	0.446^1^
	Control	35	7.06 ± 1.48	6.6 (5.17–11.31)	35	7.06 ± 1.48	6.6 (5.17–11.31)	
	*p*-value	0.019^2^				0.014^2^		
NLR	Case	35	3.85 ± 6.93	2.12 (1.19–4.73)	35	2.24 ± 0.85	1.81 (1.34–33.54)	0.931^1^
	Control	35	1.85 ± 0.66	1.84 (0.83–3.66)	35	1.85 ± 0.66	1.84 (0.83–3.66)	
	*p*-value	0.093^2^				0.129^2^		
PLR	Case	35	117.48 ± 37.99	110.13 (53.9–202.19)	35	117.64 ± 34.26	112.97 (60.9–206.38)	0.88^1^
	Control	35	120.18 ± 39.77	114.49 (53.7–247.65)	35	120.18 ± 39.77	114.49 (53.7–247.65)	
	*p*-value	0.757^2^				0.806^2^		
Periodontal parameters								
PI	Case	30	2.37 ± 0.21	2.45 (2.02–2.66)	30	0.95 ± 0.23	0.9 (0.65–1.45)	< .001^1^
	Control	27	0.56 ± 0.16	0.58 (0.29–0.82)	27	0.56 ± 0.16	0.58 (0.29–0.82)	
	*p*-value	< .001^2^				< .001^2^		
GI	Case	35	2.52 ± 0.19	2.48 (2.2–2.87)	35	1.38 ± 0.19	1.41 (0.86–1.64)	< .001^1^
	Control	35	0.61 ± 0.18	0.62 (0.15–0.86)	35	0.61 ± 0.18	0.62 (0.15–0.86)	
	*p*-value	< .001^2^				< .001^2^		
PD	Case	35	4.56 ± 0.6	4.52 (3.86–5.87)	35	2.54 ± 0.3	2.48 (2.12–3.2)	< .001^1^
	Control	35	1.57 ± 0.22	1.61 (1.05–1.86)	35	1.57 ± 0.22	1.61 (1.05–1.86)	
	*p*-value	< .001^2^				< .001^2^		
BOP	Case	35	72.77 ± 4.49	74.8 (62.4–78.8)	35	23.83 ± 3.18	23.95 (18.6–30.6)	< .001^1^
	Control	35	6.5 ± 1.54	6.43 (3.78–8.65)	35	6.5 ± 1.54	6.43 (3.78–8.65)	
	*p*-value	< .001^2^				< .001^2^		
CAL	Case	35	5.12 ± 0.47	5.13 (4.44–6.59)	35	2.72 ± 0.34	2.65 (2.2–3.4)	< .001^1^
	Control	35	0 ± 0	0 (0–0)	35	0 ± 0	0 (0–0)	
	*p*-value	< .001^2^				< .001^2^		

*CRP* C-reactive protein, *Prc* procalcitonin, *IG* immature granulocytes, *DNI* delta neutrophil index, *Neu* neutrophil count, *Lym* lymphocyte, *Plt* platelet, *WBC* white blood cell, *NLR* neutrophil–lymphocyte ratio, *PLR* thrombocyte-lymphocyte ratio, *PI* plaque index, *GI* gingival index, *PD* pocket depth, *BOP* bleeding on probing, *CAL* clinical attachment level

^1^Wilcoxon signed-rank test

^2^Mann-Whitney *U* test

**Fig. 1:**
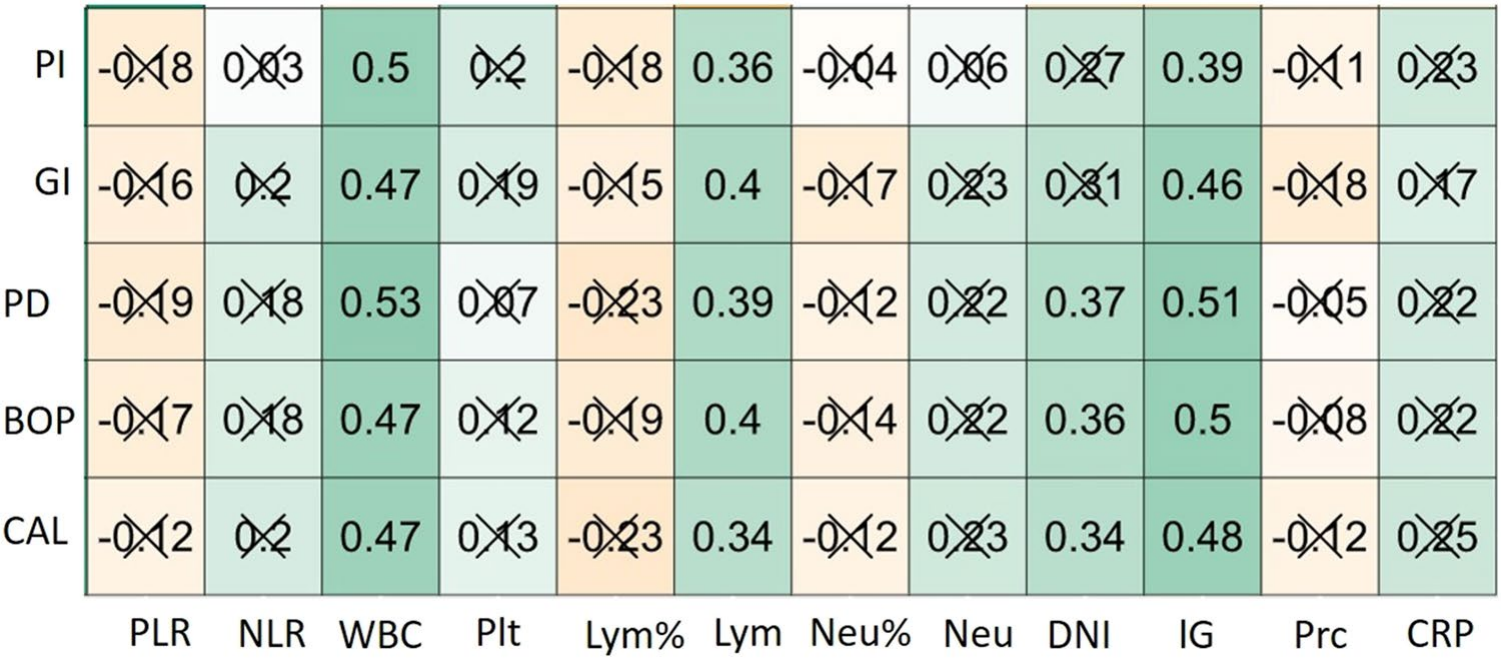
Evaluation of the correlation between periodontal and inflammatory variables 3 months after NSPT in the periodontitis group. Correlation matrix boxes: green and orange boxes indicate positive and negative correlations, respectively (color tones indicate the strength of the correlation). Boxes without “X” indicate significance (*p* < 0.05). The numbers on the boxes are Spearman correlation coefficients. CRP (C-reactive protein), Prc (procalcitonin), IG (immature granulocytes), DNI (delta neutrophil index), Neu (neutrophil count), Lym (lymphocyte), Plt (platelet) WBC (white blood cell), NLR (neutrophil-lymphocyte ratio), PLR (thrombocyte-lymphocyte ratio)

The relationships between the periodontal and inflammatory variables in the test group are shown in Fig. 1.PI was significantly positively correlated with WBC (*r* = 0.5), Lym (0.36), and lG (0.39) (*p* < 0.05). In addition, GI was significantly positively correlated with WBC (*r* = 0.47), Lym (0.4), and lG (0.46) (*p* < 0.05). Regarding SCD, there was a significant positive correlation with WBC (*r* = 0.53), Inf (0.39), DNI (0.37), and lG (0.51) (*p* < 0.05). Between BOP and WBC (*r* = 0.47), Lym (*r* = 0.4), DNI (*r* = 0.36), and IG (*r* = 0.5), significant positive correlations were observed (*p* < 0.05). In the last, also significant positive correlations were found between CAL and WBC (*r* = 0.47), Lym (*r* = 0.34), DNI (*r* = 0.34), and IG (*r* = 0.48) (*p* < 0.05). No significant correlation was observed in other comparisons (*p* > 0.05).

## Discussion

The aim of this study was to examine the change in serum biomarkers and DNI and IG levels at 3 months after NPST in patients with stage 3, grade A periodontitis. At the end of the study, the null hypothesis was accepted because it was seen that there was a decrease in DNI values after NPST, but this decrease was not significant.

In patients with periodontitis, bacterial components or locally produced pro-inflammatory cytokines can enter the circulation and can cause systemic inflammation [28]. The therapeutic targets of periodontal treatment are to prevent disease progression by reducing or eliminating pathogens and metabolites, to maintain oral health, comfort, and function with appropriate aesthetics, and ultimately to prevent the recurrence of periodontitis. NSPT aims to reduce the number of periodontal pathogens and thereby reduce inflammation. As a result of this study, there was seen to be a close relationship between the initial DNI value and periodontal disease. After periodontal treatment, the DNI values were found to have decreased, but this decrease was not significant. In a previous cross-sectional study in 2022 by the same authors, the relationship between DNI and periodontal disease was examined for the first time [21]. In that study, the DNI and IG values were seen to be significantly higher in patients with gingivitis and periodontitis compared to a healthy control group [21]. To our knowledge, our study is the first to evaluate the effect of periodontal treatment on DNI and IG values.

Inflammatory mediators released because periodontitis can stimulate the production of CRP from hepatocytes. Mediators such as tumor necrosis factor alpha, interleukin-6 and interleukin-1 in particular, function in this process [29]. In this sense, periodontal infection can lead to systemic inflammation with a significant increase in CRP levels. In the samples taken at the start of the current study, the CRP level was observed to be significantly higher in the patients with periodontitis compared to those without periodontitis. Karattil et al. [8] showed that there was a significant difference in CRP levels as the severity of periodontal disease progressed. Glurich et al. [9] reported a significant increase in CRP levels in patients with > 4 mm attachment loss and BOP. By strengthening the theory that periodontitis has a significant effect on inflammatory biomarker levels, this positive relationship suggested that periodontal infection could lead to a systemic effect and could support the development and exacerbation of other pathologies.

In almost all the studies examining the relationship between periodontitis and serum CRP levels, it has been emphasized that the individuals evaluated had systemic diseases such as diabetes, cardiovascular diseases, or rheumatoid arthritis [29]. The presence of systemic variables could be a confounding factor in the evaluation of the effect of periodontitis on CRP levels. Therefore, as only systemically healthy individuals were included in the current study, this confounding factor was eliminated. However, it is possible that there was the limitation of some unknown systemic changes in the samples of some individuals. The findings of whether periodontal treatment has an effect on CRP levels seem to be inconsistent [30, 31]. There are studies in the literature showing a significant decrease in CRP level after periodontal treatment, and there are also studies showing no change [30]. The reason for this can be stress or the presence of an undiagnosed inflammatory condition in the individual.

Periodontitis can create systemic effects through pathological changes caused by leukocytes [5]. Thrombocytes are an important component of blood and closely associated with inflammation. When activated, pro-inflammatory mediators are released, and pro-inflammatory receptors emerge. This causes thrombocytes to bind to WBC and endothelial cells. Pathogens located in periodontal tissues can easily stimulate thrombocytes and WBC [5]. Like the current study results, Ustaoğlu et al. showed that WBC and neutrophil counts were higher in a periodontitis group than in a control group, and following NSPT, the WBC count reduced [5]. It was seen that periodontitis increased the WBC count, and after treatment, the elevated WBC count decreased. This is consistent with the view that periodontitis causes reversible systemic inflammation. Periodontitis treatment resulting in resolution of the local inflammatory response may be able to alleviate chronic cellular inflammatory changes.

In recent studies, NLR and PLR, which are defined as the ratio of absolute neutrophil or thrombocyte and lymphocyte counts, have been suggested as effective biomarkers in the prognosis of several inflammatory diseases [32]. In patients with periodontitis and systemic diseases, the NLR has been reported to be increased. The NLR value has been shown to be higher in periodontitis patients with hyperlipemia compared to those without [33]. Torrungruang et al. [34] reported that the NLR value was related to the severity of periodontitis in patients with diabetes, but there was no correlation with the glycemic status. PLR was determined to be related to both the severity of periodontal disease and the glycemic status. Therefore, by establishing a bridge between periodontal and systemic conditions, NLR and PLR may serve as potential biomarkers of the systemic inflammatory response to chronic periodontitis [7]. Acharya et al. reported a positive correlation between chronic periodontitis and NLR and PLR and determined that both values decreased after NSPT [7]. However, other studies have shown conflicting results [32]. In a recent study, NLR was seen to be associated with inflammation and disease severity in patients with generalized aggressive periodontitis, but PLR did not show a similar effect [32]. Moreover, in another study, no significant differences were found in NLR and PLR values in the presence of periodontitis and gingivitis [21]. In the current study, systemically healthy patients with stage 3, grade A periodontitis were included, and although an increase was observed in the NLR and PLR values in the periodontitis group, the difference was not statistically significant. Thus, it can be said that this parameter is not a sufficiently sensitive and specific biomarker for a patient group with low-level chronic inflammation, such as periodontitis.

In a review by Park et al. [35] it was stated that DNI was a useful diagnostic tool in infected patients, could predict mortality in these patients, and should be used more widely in clinical practice. In another study, DNI was determined to be a predictor of prognosis in patients with chronic obstructive pulmonary disease, and the increase in DNI at advanced stages of the disease was significant [35]. Ahn et al. [36] showed a positive correlation between DNI and bacteremia in children with immune failure. Despite various reports that DNI is a marker that can be used in the diagnosis of infection, there are insufficient studies in literature about whether DNI is valid in the diagnoses of chronic inflammatory diseases or whether the diagnostic accuracy of DNI is comparable to that of other markers. There is only one study in the literature that has examined the relationship between DNI and periodontal diseases [21]. In that study, published in 2022, DNI levels were determined to be increased in patients with periodontitis and gingivitis, and it was stated that DNI could be a new biomarker for periodontal diseases. This is supported by the results of the current study. The DNI values in the current study decreased after NSPT, but this change was not determined to be statistically significant. The decrease in DNI level can be thought to be due to the treatment of periodontal inflammation. However, as the change was not at a statistically significant level, this could have been due to the DNI level having been affected by several factors such as potentially unknown chronic infections and/or inflammatory conditions in the patient, age, and trauma. Therefore, there is a need for further studies with larger patient populations to be able to evaluate the effect of periodontal treatment on DNI.

Bacteria and their products cause bone destruction in periodontal diseases. Chemotactic factors and cytokines activated because periodontitis stimulates the expression of IG from the bone marrow into circulation [37]. The increase in the DNI value in the current study could be a result of this. Obtaining the biomarker of DNI is relatively simpler and lower cost than other blood biomarkers that require more complex laboratory tests [35]. In addition, the half-life of DNI is 3 h, which is a shorter period than the 24–30 h of procalcitonin [38]. A shorter half-life more easily reflects the infection status and is helpful in explaining the therapeutic efficacy of treatment during follow-up. In the current study, while no difference was seen in the procalcitonin levels between the groups, the difference between the groups with respect to DNI was determined to be statistically significant. This suggests that DNI could be a more effective predictor of the level of systemic inflammation than procalcitonin. Park et al. compared the prognostic and predictive values of DNI and procalcitonin biomarkers with ROC curve analysis and determined that the diagnostic accuracy of DNI was better [39].

There were some limitations to this study, primarily that although power analysis was performed and the number of patients seemed to be sufficient, comparisons between groups with greater patient numbers could provide more meaningful results. In addition, by making the postoperative evaluations more frequently, the short-term effects of periodontal treatment on DNI and IG could be examined in more detail. A further limitation was that the effect of periodontal disease on the DNI values was only examined in serum samples, and it could be more useful to make examinations of different body fluids such as saliva and gingival groove fluid. However, in the control group, blood samples were not taken after 3 months, considering that they were systemically and periodontally healthy individuals. However, inflammatory disorders that the person is not even aware of can affect serum DNI values [16, 19, 27]. Therefore, in future studies, serum and blood samples from the control group can be repeated and evaluated during follow-up periods. In addition, the correlation between the effect of NPST on serum DN and IG values and pro-inflammatory markers in GCF can be examined.

## Conclusion

This study is the first to have evaluated the effect of NSPT on DNI levels in patients with periodontitis. Consistent with previous findings in the literature, the patients with periodontitis were determined to have higher levels of DNI, CRP, neutrophils, and WBC, compared to the individuals without periodontitis. Although a decrease was seen in DNI after NSPT, this was not at a significant level. Although these markers are guidance in the evaluation of inflammation at the start of periodontal disease, there is a need for further studies with greater numbers of cases to be able to use these parameters in the evaluation of treatment success.

### Supplementary Information

Below is the link to the electronic supplementary material.

Supplementary file1 (DOCX 34 KB)

## Data Availability

Data and materials are available at the Periodontology Department in the Faculty of Dentistry, Kahramanmaraş Sütçü Imam University.
